# Relationship between childhood abuse and substance misuse problems is mediated by substance use coping motives, in school attending South African adolescents

**DOI:** 10.1016/j.drugalcdep.2018.10.009

**Published:** 2019-01-01

**Authors:** Lee Hogarth, Lindi Martin, Soraya Seedat

**Affiliations:** a School of Psychology, University of Exeter, Washington Singer Building, Perry Road, Exeter, EX4 4QG, UK; b Department of Psychiatry, Stellenbosch University, Private Bag X1, Matieland, 7602, Stellenbosch, South Africa

**Keywords:** Childhood abuse, Substance use coping motives, Alcohol dependence, Drug use problems, Mediation analysis

## Abstract

**Background:**

In adults, it has been shown that the relationship between childhood abuse and substance misuse problems is mediated by the belief that substance use helps cope with negative affective states. By contrast, in adolescents, it is unknown whether drug use coping motives play this same mediating role.

**Methods:**

Secondary analysis of 1149 school attending adolescents in Cape Town, South Africa (average age = 16.24 years, range = 13–23; 60% female). Questionnaire measures obtained during a single test session (among a larger battery) assessed childhood trauma (CTQ), alcohol (AUDIT) and drug problems (DUDIT), and coping orientation (A-COPE) which contained three items assessing drug use to cope with negative affect.

**Results:**

The three types of childhood abuse measured by the CTQ – emotional, physical and sexual – were positively associated with greater alcohol/drug problems, and drug use coping motives. Drug use coping motives mediated the relationships between childhood abuse types and alcohol/drug problems, and these mediational pathways remained significant when gender and other subscales of the A-COPE were included as covariates.

**Conclusions:**

These data are preliminary insofar as drug use coping motives were assessed with a non-validated subscale of the A-COPE. Nevertheless, drug use to cope with negative affect mediated the relationship between all three types of childhood abuse (emotional, physical, sexual) and alcohol/drug problems in school attending adolescents. The implication is that drug prevention programs for this risk group should seek to mitigate drug use coping motives.

## Highlights

• Child abuse linked to drug problems in South African school attending adolescents.
• Drug use coping motives mediated this relationship.
• Prevention programs should target drug use coping motives.

## 1 Introduction

Childhood abuse is typically associated with greater risk of alcohol and drug problems, in cross sectional and prospective designs, in adolescent and adults, although not all studies replicate this finding (Brady and Back, 2012; Butt et al., 2011; Konkolÿ Thege et al., 2017; Simpson and Miller, 2002). Childhood abuse/trauma (assessed in adults) is also associated with stronger drug use coping motives, which statistically mediates the relationship between abuse/trauma and alcohol/drug problems (Goldstein et al., 2010; Grayson and Nolen-Hoeksema, 2005; Schuck and Widom, 2001; Vilhena-Churchill and Goldstein, 2014). Similarly, the relationship between adult traumata (e.g., partner violence, sexual coercion, etc.) and drug/alcohol problems is mediated by drug use coping motives (Asberg and Renk, 2012; Fossos et al., 2011; Kaysen et al., 2007; Øverup et al., 2015; Ullman et al., 2005). Finally, the relationship between adult psychiatric states (including depression and anxiety) and alcohol/drug problems is mediated by drug use coping motives (Dvorak et al., 2014; Gonzalez et al., 2011; Holahan et al., 2001; McDevitt-Murphy et al., 2015; Mooney et al., 2008;O’Hare and Sherrer, 2011; Peirce et al., 1994; Reardon et al., 2002; Schuckit et al., 2006; Simpson et al., 2014; Stewart et al., 2001; Yeater et al., 2010; Young-Wolff et al., 2009). These studies suggest drug use coping motives are a crucial psychological mechanism driving substance dependence in vulnerable groups.

It is currently unknown whether, in school attending adolescents, drug use coping motives mediate the relationship between childhood abuse and alcohol/drug problems, as the forgoing mediation studies have all recruited adults. Indeed, there is a more general question regarding the relative importance of coping motives (as opposed to other substance use motives) as a risk factor for alcohol/drug in the young (Kuntsche et al., 2005). One study, however, does suggest that coping motives are an important risk factor for bullied adolescents (Topper et al., 2011). This study recruited school attending British children aged 13–15, and found that the prospective relationship between being bullied and alcohol problems at one year follow-up was mediated by drinking to cope with negative affect at follow-up, even when other drinking motives were statistically controlled. It remains to be seen whether coping motives mediate the relationship between child abuse and substance problems in adolescents, specifically.

South African adolescents, and the South African population in general, report high rates of childhood abuse/trauma and alcohol/drug problems (Kaminer et al., 2013a, b; Parry et al., 2004; Stansfeld et al., 2018; Stein et al., 2008; Suliman et al., 2009). Furthermore, childhood abuse/trauma is positively associated with alcohol/drug problems in these samples (Morojele et al., 2016; Saban et al., 2014; Sommer et al., 2017) raising questions as to whether this relationship is mediated by coping motives. To address this question, the current study conducted secondary analysis of an existing data set collected to explore the relationship between childhood abuse and psychiatric symptoms in school attending South African adolescents (Martin et al., 2014). The measures relevant to the current hypothesis were the Childhood Trauma Questionnaire (CTQ, Bernstein et al., 2003), the Alcohol and Drug Use Disorders Identification Tests (AUDIT, Babor et al., 2001, and DUDIT, Berman et al., 2005). Crucially, the Adolescent Coping Orientation for Problem Experiences questionnaire (A-COPE, Patterson and McCubbin, 1987) was also recorded, originally to examine protective factors (the ACOPE has been validated with South African Adolescents (Spangenberg and Henderson, 2001). However, the A-COPE includes three items that assess whether individuals use alcohol, smoking or drugs to cope with problems. Although this subscale of coping motives has not previously been validated, the items are descriptively similar to existing coping motives measures (e.g., Grant et al., 2007), and single item assays of coping motives have previously served as excellent prospective markers for future alcohol problems (Crum et al., 2013; Menary et al., 2011). In the current analysis, if drug use coping motives indexed by the A-COPE mediate the relationship between childhood abuse (CTQ) and alcohol/drug problems (AUDIT/DUDIT), these mediational paths would suggest that drug prevention programs for vulnerable adolescents should focus on mitigating coping motives specifically – a therapeutic approach that is becoming more widely utilised, and has demonstrated efficacy in other vulnerable populations (Anker et al., 2016; Bradizza et al., 2017; Conrod et al., 2013; Stasiewicz et al., 2013; Wurdak et al., 2016).

## 2 Method

### 2.1 Participants

Participants were 1149 school attending adolescents from a representative sample of secondary schools (n = 29) in Cape Town, South Africa. Mean age was 16.24 years (range = 13–23, SD = 1.95), there were 59.97% girls (689/1149), 68.9% identified themselves as Black (792/1149), and mean education level was grade 9 (range = 8–12, SD = 1.30).

### 2.2 Procedure

Access to secondary schools was granted by the Western Cape Education Department. Ethical approval was provided by Stellenbosch University Health Research Ethics Committee. Randomly selected Cape Town secondary schools were approached. Schools that participated provided names of learners from grades 8 to 12, from which 20 learners per grade were randomly selected. Written consent was obtained from parents/guardians and written assent was obtained from learners. Questionnaires were completed within schools on a single occasion.

### 2.3 Questionnaires

The Childhood Trauma Questionnaire (CTQ, Bernstein et al., 2003) is a 28-item retrospective self-report measure of the frequency of and severity of emotional, physical and sexual abuse. The CTQ also contains emotional and physical neglect, and minimisation/denial subscales. This paper only reported the abuse subscales for simplicity. Participants responded to each item in the context of “When I was growing up” and answered according to a 5-point Likert scale ranging from ‘never true’ (1) to ‘very often true’ (5). The abuse subscales were each summed across five items, and scores ranged from 5 to 25. Severity groups within each abuse type were defined as ‘none’, ‘low, ‘moderate’ and ‘severe’, using pre-established score boundaries (Bernstein et al., 2003): Emotional Abuse (5–8, 9–12,13-15 and 16+); Physical Abuse (5–7; 8–9; 10–12; 13+); Sexual Abuse (5; 6–7; 8–12; 13+). The Cronbach’s alpha of the three CTQ subscales were .72, .77 and .80 respectively (see Table 1B) indicating acceptable internal consistency of the items.

The Alcohol Use Disorders Identification Test (AUDIT, Babor et al., 2001) is a 10–item questionnaire measuring alcohol use problems (total score range 0–40). Scores between 0–7 indicate low-risk alcohol use, whereas scores of 8+ indicate hazardous drinking. The Cronbach’s alpha of the AUDIT was .87 indicating good internal consistency of the items.

The Drug Use Disorders Identification Test (DUDIT, Berman et al., 2005) is an 11-item questionnaire measuring drug use problems (total score range 0–44). For males, scores of 6+, and for females, scores of 2+ are taken to indicate problematic drug use. The Cronbach’s alpha of the DUDIT was .89 indicating good internal consistency of the items.

Adolescent Coping Orientation for Problem Experiences (A-COPE, Patterson and McCubbin, 1987) is a 54-item questionnaire measuring coping strategies, endorsed in the context of the question ‘When you face difficulties or feel tense, how often do you.’ Each coping strategy was endorsed on a 5-point Likert scale labelled ‘Never’ (1), ‘Hardly’ (2), ‘Sometimes’ (3), ‘Often’ (4) and ‘Most of the time’ (5). Three items assessed the use of drugs to cope: Item 24: ‘Use drugs (not necessarily prescribed by a doctor)’; Item 42: ‘Smoke’; Item 46: ‘Drink beer, wine, liquor.’ These items were averaged to create a bespoke composite index of drug use coping motives, descriptively similar to existing validated assays of coping motives (e.g., Grant et al., 2007) and single item measures of coping motives (Crum et al., 2013; Menary et al., 2011). Scores of 3+ (average across items) were considered clinically meaningful insofar as they indicate that the participant (on average) endorsed using drugs to cope at least ‘Sometimes.’ The Cronbach’s alpha of the A-COPE bespoke drug coping subscale was .54 indicating poor internal consistency of the items (see Discussion). The A-COPE has 12 standard subscales (ventilating, diversions, self-reliance, social support, solving family problems, avoiding problems, spiritual, close friends, professional support, demanding activity, being humorous and relaxing), which were treated as covariates apart from the Avoiding Problems subscale, which encompasses the three drug coping items.

### 2.4 Data analysis

**Fig. 1:**
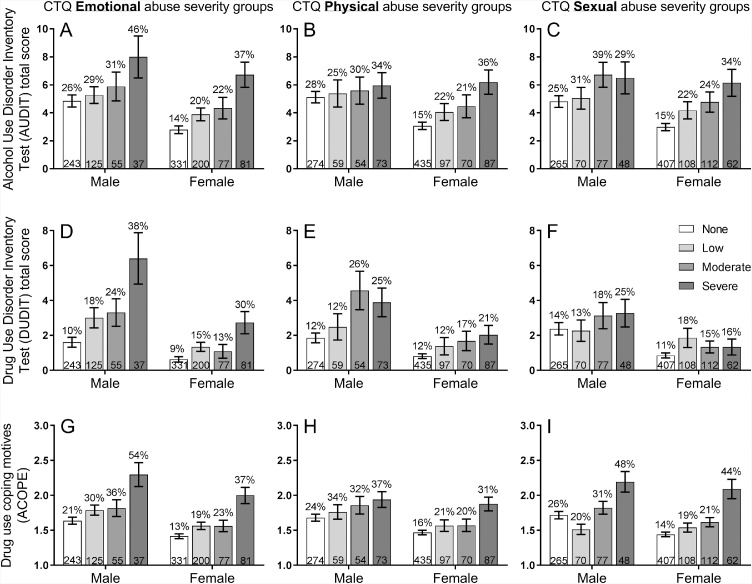
In Fig. 1A–I, participants were group-ed based on the severity of abuse reported in the CTQ Childhood Trauma Questionnaire, into none, low, moderate and severe groups, separately for each gender. The three columns report emotional, physical, sexual abuse respectively. The three rows report the mean (and SEM) of alcohol problems (AUDIT), drug problems (DUDIT), and drug use coping motives (A-COPE), respectively. For each pane (A–I), a separate ANOVA was conducted with the drug measure (AUDIT, DUDIT or A-COPE) as the dependent variable, and the between-subject variables CTQ severity group and gender. Adolescents in the severe emotional, physical and sexual abuse categories showed higher rates of alcohol and drug problem, and drug use coping motives (apart from sexual abuse which was not associated with greater drug problems in the DUDIT, Fig. 1F). These relationships between childhood abuse and alcohol/drug measures did not differ between genders (apart from the relationship between emotional abuse and drug problems which was stronger in males - Fig. 1D). The percentage value shown above each bar is the proportion of each CTQ abuse severity group that reported a ‘clinically meaningful’ level of the drug outcome: AUDIT = hazardous drinking (8+ scores); DUDIT = drug problems (6+ for males, 2+ for females); A-COPE = drug use coping score of 3+ indicating that the participant (on average across items) endorsed using drugs to cope at least ‘Sometimes’. The numbers at the bottom of each bar is the N of participants falling into each CTQ abuse severity group.

In Fig. 1, A–I, participants were grouped based on the severity of abuse reported in the CTQ Childhood Trauma Questionnaire, into none, low, moderate and severe groups, separately for each gender. The three columns report emotional, physical, sexual abuse respectively. The three rows report the mean (and SEM) of alcohol problems (AUDIT), drug problems (DUDIT), and drug use coping motives (A-COPE), respectively. For each pane (A–I), a separate ANOVA was conducted with the drug measure (AUDIT, DUDIT or A-COPE) as the dependent variable, and the between-subject variables CTQ severity group and gender. A significant main effect of CTQ severity group would indicate that the form of abuse was related to the drug outcome (irrespective of gender). A significant main effect of gender would indicate that the drug outcome differed between genders. Finally, a significant interaction between CTQ severity group and gender would indicate that the form of abuse was more strongly related to the drug outcome in one gender. Where the main effect of CTQ severity group was significant for a pane, Games-Howell post hoc tests were used to compare every pair of CTQ groups.

Mediation models were tested with 5000 bias corrected bootstrapped confidence intervals, using Hayes and colleagues (Preacher and Hayes, 2004) software within SPSS (all other statistics were in SPSS). This method was favoured because it arguably produces the least Type I and Type II errors (Preacher and Hayes, 2008), and because it is thought to have greater power to detect mediational effects than alternative approaches (MacKinnon et al., 2002). None of the variables used in the analysis were normally distributed, the CTQ abuse groups did not have homogenous variance in AUDIT, DUDIT or drug use coping scores, and there were unequal Ns for CTQ abuse severity groups. ANOVA, Hayes’ bootstrapping method, and Games-Howell post hoc tests are considered robust against these parameters.

## 3 Results

Fig. 1 paneA–I show the relationships between three childhood abuse types separated into columns (physical, emotional, sexual) and the three drug outcome measures separated into rows (AUDIT, DUDIT and drug use coping motives). Inspection of Fig. 1 indicates that adolescents in the severe emotional, physical and sexual abuse categories showed higher rates of alcohol and drug problems, and drug use to cope with negative affect (the one exception is sexual abuse which was not associated with greater drug problems on the DUDIT, Fig. 1F), and these relationships did not differ between genders (but see Fig. 1D).

### 3.1 Relationship between childhood abuse and alcohol problems

Following the analytical plan outlined above, ANOVA on Fig. 1A produced a significant main effect of emotional abuse severity, *F*(3,1141) = 9.09, *p* < .001, η_p_^2^ = .023, indicating that emotional abuse was associated with increased alcohol problems across genders. There was also a significant main effect of gender, *F*(1,1141) = 10.35, *p* = .001, η_p_^2^ = .009, indicating that males had more alcohol problems. However, there was no significant interaction, *F*<1, indicating that the association between emotional abuse and alcohol problems was comparable between genders. Post hoc tests indicated that the severe group differed from the none, *p* < .001, and low group, *p* = .01, but not the moderate group, *p* = .14. ANOVA on Fig. 1B produced significant main effects of physical abuse severity, *F*(3,1141) = 4.26, *p* = .005, η_p_^2^ = .011, and gender, *F*(1,1141) = 4.71, *p* = .03, η_p_^2^ = .004, but no interaction, *F*(3,1141) = 1.38, *p* = .25, η_p_^2^ = .004. Only the severe and none groups differed, *p=*.005. ANOVA on Fig. 1C produced significant main effects of sexual abuse severity, *F*(3,1141) = 6.94, *p* < .001, η_p_^2^ = .018, and gender, *F*(1,1141) = 6.69, *p* = .01, η_p_^2^ = .006, but no interaction, *F*<1. Only the severe and none groups differed, *p<*.005.

### 3.2 Relationship between childhood abuse and drug problems

ANOVA on Fig. 1D produced significant main effects of emotional abuse severity, *F*(3,1141) = 17.83, *p* < .001, η_p_^2^ = .045, and gender, *F*(1,1141) = 39.28, *p* < .001, η_p_^2^ = .033, and a significant interaction, *F*(3,1141) = 2.85, *p* = .037, η_p_^2^ = .007. In each gender, only the severe group differed from the none group, *p*s≤.014. ANOVA on Fig. 1E produced significant main effects of physical abuse severity, *F*(3,1141) = 9.18, *p* < .001, η_p_^2^ = .024, and gender, *F*(1,1141) = 24.95, *p* < .001, η_p_^2^ = .021, but no interaction, *F*(3,1141) = 1.59, *p* = .19, η_p_^2^ = .004. Only the severe and none groups differed, *p=*.005. ANOVA on Fig. 1F produced no significant main effects of sexual abuse severity, *F*(3,1141) = 1.44, *p* = .23, η_p_^2^ = .004, (indicating no relationship between sexual abuse and drug problems), a significant main effect of gender, *F*(1,1141) = 16.91, *p* < .001, η_p_^2^ = .015, and no interaction, *F*<1.

### 3.3 Relationship between childhood abuse and drug use coping motives

ANOVA on Fig. 1G produced significant main effects of emotional abuse severity, *F*(3,1141) = 19.16, *p* < .001, η_p_^2^ = .048, and gender, *F*(1,1141) = 18.51, *p* < .001, η_p_^2^ = .016, but no interaction, *F*<1. The severe group differed from all the remaining groups, *p*s≤.003. ANOVA on Fig. 1H produced significant main effects of physical abuse severity, *F*(3,1141) = 8.16, *p* < .001, η_p_^2^ = .021, and gender, *F*(1,1141) = 10.31, *p* = .001, η_p_^2^ = .009, but no interaction, *F*<1. The severe group differed from the none group, *p* < .001, and low group, *p* = .037. ANOVA on Fig. 1I produced significant main effects of sexual abuse severity, *F*(3,1141) = 18.48, *p* < .001, η_p_^2^ = .046, and gender, *F*(1,1141) = 5.91, *p* = .015, η_p_^2^ = .005, but no interaction, *F*(3,1141) = 1.85, *p* = .14, η_p_^2^ = .005. The severe group differed from all the other groups, *p*s≤.001.

### 3.4 Mediation analysis

Table 1A shows six mediation models with CTQ childhood abuse types (emotional, physical, sexual) as predictors (X), alcohol (AUDIT) or drug (DUDIT) problems as the outcome (Y), and drug use coping motives (A-COPE) as the mediator (M). The beta coefficients of the component paths (a, b, c) indicate that there were significant interrelationships between the CTQ abuse types, drug use coping motives and alcohol/drug problems (confirmed by the correlation matrix shown in Table 1B). The one exception was CTQ sexual abuse which was not related to drug use problems in the DUDIT (c path total effect; see also Table 1B and Fig. 1F). Most importantly, the indirect mediation paths were all significant (the 95% confidence intervals do not encompass zero). Finally, the c' paths between X→Y remained significant when the mediational paths were controlled for in four models, indicating partial mediation, whereas the c' path was not significant for models with sexual abuse as the predictor, indicating full mediation. The mediational paths remained significant when gender and the 11 subscales of the A-COPE were entered as covariates into the models, suggesting the mediational role of drug use coping motives could not be explained by other coping styles.

**Table 1A:** Mediation models. Unstandardized beta coefficients (β) and 95% confidence intervals (CI) from mediational models calculated using Heyes Process bias corrected bootstrap method (5000 samples). *p < .05, **p < .01, ***p < .001. CTQ = Childhood Trauma Questionnaire, abuse subscales. A-COPE = Adolescent Coping Orientation for Problem Experiences, bespoke subscale assessing drug, smoking and alcohol use to cope. AUDIT = Alcohol Use Disorders Identification Test. DUDIT = Drug Use Disorders Identification Test. The a, b, and c paths indicate that there were significant interrelationships between CTQ abuse types (emotional, physical, sexual), A-COPE drug use coping motives, and alcohol/drug problems indexed by the AUDIT/DUDIT. The one exception is that CTQ sexual abuse was not related to drug use problems in the DUDIT (c path). All six indirect mediational paths were significant (the 95% confidence intervals do not encompass zero) demonstrating that coping motives mediate the relationship between childhood abuse and alcohol/drug problems. The direct effect (c' paths) between X→Y remained significant when the mediational paths were controlled for in four models, indicating partial mediation, whereas the direct effect was not significant for models with sexual abuse as the predictor indicating full mediation. The overall conclusion is that drug use coping motives mediate the relationship between childhood abuse and alcohol/drug problems in school attending South African adolescents.

			Component paths			Mediation	
X (predictor)	M (mediator)	Y (outcome)	*a* path: X→M	*b* path: M→Y controlling for X	*c* path: total effect X→Y	*c*' path: direct effect X→Y controlling for the indirect mediational path	Indirect mediational path: X→M→Y
CTQ Emotional abuse score	A-COPE drug use coping motives	AUDIT total	β = .04, CI = .03- .05***	β = 4.09, CI = 3.67-4.52***	β = .26, CI = .17-.35***	β = .09, CI = .02-.17*	β = .17, CI = .12-.22***
CTQ Physical abuse score			β = .04, CI = .02- .05***	β = 4.12, CI = 3.70-4.54***	β = .24, CI = .15-.33***	β = .10, CI = .02-.18*	β = .14, CI = .09-.20 ***
CTQ Sexual abuse score			β = .05, CI = .03- .06***	β = 4.17, CI = 3.74-4.59***	β = .24, CI = .13-.34***	β = .04, CI=-.05-.13	β = .19, CI = .13-.26 ***
CTQ Emotional abuse score		DUDIT total	β = .04, CI = .03- .05***	β = 2.56, CI = 2.25-2.87***	β = .19, CI = .13-.25***	β = .09, CI = .03-.14*	β = .10, CI = .07-.14 ***
CTQ Physical abuse score			β = .04, CI = .02- .05***	β = 2.58, CI = 2.27-2.89***	β = .18, CI = .11-.24***	β = .09, CI = .03-.14**	β = .09, CI = .06-.13 ***
CTQ Sexual abuse score			β = .05, CI = .03- .06***	β = 2.72, CI = 2.41-3.03***	β = .06, CI = .-01-.14	β=-.06, CI=-.13-.01	β = .13, CI = .08-.18***

**Table 1B:** Pearson correlation coefficients (*r*) between CTQ abuse types (emotional, physical, sexual), drug use to cope and alcohol/drug problems. The Cronbach’s alpha of subscales is shown on the diagonal in brackets.

	1	2	3	4	5	6
1. CTQ emotional abuse score	(.72)					
2. CTQ Physical abuse score	*r*=.60 ***	(.77)				
3. CTQ Sexual abuse score	*r*=.38 ***	*r*=.42 ***	(.80)			
4. A-COPE Drug use coping motives	*r*=.22 ***	*r*=.18 ***	*r*=.21 ***	(.54)		
5. AUDIT total	*r*=.17 ***	*r*=.15 ***	*r*=.13 ***	*r*=.51 ***	(.87)	
6. DUDIT total	*r*=.18 ***	*r*=.16 ***	*r*=.05	*r*=.45 ***	*r*=.36 ***	(.89)

## 4 Discussion

The main finding was that childhood abuse types (emotional, physical, sexual) were associated with increased alcohol/drug problems, and drug use coping motives. The exception was the relationship between sexual abuse and drug problems, which was not significant. These findings corroborate the majority of studies which have demonstrated that childhood abuse is associated with substance problems (Brady and Back, 2012; Butt et al., 2011; Konkolÿ Thege et al., 2017; Simpson and Miller, 2002). There were no differences between the genders in the strength of these associations, apart from emotional abuse, which had a stronger relationship with drug problems in males (Fig. 1D). Post hoc contrasts suggested that severe abuse was associated with a jump in alcohol/drug problems and coping motives, and that there was no significant linear increase across the less severe abuse groups despite the numerical trend. The implication is that individuals who fall into the severe abuse group could be selected for prevention interventions. Importantly, the relationships between childhood abuse and alcohol/drug problems were demonstrated in a non-western sample, providing rare cross-cultural corroboration (Konkolÿ Thege et al., 2017).

The most important finding was that drug use coping motives mediated the relationship between childhood abuse and alcohol/drug problems. These mediational pathways remained significant when gender and other subscales of the A-COPE were entered as covariates suggesting that the mediating role of drug use coping motives could not be explained by other coping styles. The study is the first to confirm this mediational pathway in school attending adolescents (it has previously only observed in adults), and in South African adolescents, suggesting the mediational path is found cross culturally. Such cross-cultural generalisation may have been doubted given that previous studies with South African university students found that coping motives were endorsed less than enhancement motives (Maphisa and Young, 2018; Peltzer, 2003; Gire, 2002) suggesting that coping motives might be less pronounced in South Africa (but see Kuntsche et al., 2014). The current study suggests that because of their mediating role, drug use coping motives should be targeted by drug prevention programs designed for abused/traumatised South African Adolescents specifically, but also for other vulnerable groups where coping motives play the same mediating role (Anker et al., 2016; Bradizza et al., 2017; Conrod et al., 2013; Stasiewicz et al., 2013; Wurdak et al., 2016).

The main limitation was the non-validated index of drug use coping motives derived from three items of the A-COPE. Although these items are descriptively similar to validated questionnaires of coping motives (e.g., Grant et al., 2007) and single item assays of coping motives which have previously served as excellent prospective markers for future alcohol problems (Crum et al., 2013; Menary et al., 2011), the Cronbach’s alpha for these three items was .54, indicating poor internal consistency. However, Cronbach’s alpha decreases with fewer items so a lower threshold for acceptability is sometimes applied in such cases (Heo et al., 2015). Furthermore, the corrected item total correlations for the smoke, drugs and drink coping items were .44, .32, and .32 respectively, indicating that each item was correlated with a composite of the remaining items, and all are above the desirable cutoff of .3 (Ferketich, 1991). Finally, if each of the individual items was deleted, the overall Cronbach’s alpha of .54 would be reduced to .29, .50, and .51, respectively, indicating that each item contributed to the overall Cronbach’s alpha value. From this analysis, it might be concluded that the bespoke index of coping motives is acceptable, but minimally so, and thus evidence for the mediating role of coping motives in the current models may be deemed preliminary. A key objective of future work should be to overcome this limitation by testing whether the current mediational pathways are replicated, or ideally are stronger, when a validated, multiple-item assay of drug use coping motives is tested as the mediating variable. In addition, future studies might utilise measures of alcohol and drug problems which have been validated with adolescents specifically (Read et al., 2006) rather than the AUDIT/DUDIT which have been validated primarily with adult samples (Rumpf et al., 2013). Finally, future work should replicate the cross-sectional mediation design with a longitudinal mediation design to support the claim that drug use coping motives are causal in the growth in alcohol/drug problems in abused adolescents (Jose, 2016; Windle and Windle, 2015). These studies would strengthen calls for the development of drug prevention interventions that target drug use coping motives in abused adolescents.

## Role of the funding source

Nothing declared.

## Contributors

Hogarth undertook the secondary analysis and wrote the first draft of the paper. Martin and Seedat advised on the secondary analysis. Martin collected the data in the original study. Seedat oversaw the original study. All authors corrected the manuscript prior to submission and approved the final article.

## Conflict of interest

None declared.
